# Gaussian mixture models and semantic gating improve reconstructions from human brain activity

**DOI:** 10.3389/fncom.2014.00173

**Published:** 2015-01-30

**Authors:** Sanne Schoenmakers, Umut Güçlü, Marcel van Gerven, Tom Heskes

**Affiliations:** ^1^Donders Institute for Brain, Cognition and Behaviour, Radboud University NijmegenNijmegen, Netherlands; ^2^Institute for Computing and Information Sciences, Radboud University NijmegenNijmegen, Netherlands

**Keywords:** fMRI, reconstruction, Bayesian network, data fusion, semantic categories, unsupervised learning, probabilistic inference

## Abstract

Better acquisition protocols and analysis techniques are making it possible to use fMRI to obtain highly detailed visualizations of brain processes. In particular we focus on the reconstruction of natural images from BOLD responses in visual cortex. We expand our linear Gaussian framework for percept decoding with Gaussian mixture models to better represent the prior distribution of natural images. Reconstruction of such images then boils down to probabilistic inference in a hybrid Bayesian network. In our set-up, different mixture components correspond to different character categories. Our framework can automatically infer higher-order semantic categories from lower-level brain areas. Furthermore, the framework can gate semantic information from higher-order brain areas to enforce the correct category during reconstruction. When categorical information is not available, we show that automatically learned clusters in the data give a similar improvement in reconstruction. The hybrid Bayesian network leads to highly accurate reconstructions in both supervised and unsupervised settings.

## 1. Introduction

Machine learning techniques have made it possible to accurately encode and decode mental states from neuroimaging data. Neural encoding and decoding are topics of key importance in contemporary cognitive neuroscience. Especially visual perception has received a large amount of attention since the visual system is relatively well understood and covers a large portion of the brain. While the first decoding studies focused exclusively on the prediction of discrete states such as object category (Haxby et al., 2001) or stimulus orientation (Kamitani and Tong, 2005), more recent work has focused on the prediction of increasingly complex stimulus features, culminating in the reconstruction of the contents of perceived images (Thirion et al., 2006; Kay et al., 2008; Miyawaki et al., 2008; Naselaris et al., 2009; van Gerven et al., 2010; Güçlü and van Gerven, 2014). Striking reconstructions have been made of handwritten characters (Schoenmakers et al., 2013), faces (Cowen et al., 2014), natural images (Naselaris et al., 2009) and even the gist of video clips (Nishimoto et al., 2011). Reconstructing stimuli from the neuronal response can be done by selecting the most probable image from a prior set of images (Naselaris et al., 2009). While this leads to good results, we showed that Bayesian inversion of the neuronal response yields more accurate reconstructions for our dataset (Schoenmakers et al., 2013).

Encoding and decoding are intimately related via Bayes' rule where the probability *P*(***x***|***y***) of a stimulus ***x*** given a response ***y***, is proportional to the product of a likelihood term *P*(***y***|***x***) and a prior *P*(***x***) (Friston et al., 2008; Naselaris et al., 2010). The likelihood embodies a forward model of how the stimulus features are encoded in the neural response. The prior specifies how likely a stimulus is before observing any data. Reconstruction is then accomplished by inverse inference in a generative model, where both prior knowledge of images and the BOLD response transformed to image space contribute to the reconstructions. Thirion et al. (2006) advocated this approach to reconstruction before and it has proven to be successful. Here we expand this framework to a hybrid Bayesian network that more accurately describes the stimulus features by also incorporating higher-level semantic information. Through data fusion, the higher-level semantic information can be joined with the low-level information to obtain more accurate reconstructions. This is similar in spirit as the work of Naselaris et al. (2010). However, in contrast to that study, our approach provides an analytical solution for reconstruction.

We present a framework for image reconstruction using Gaussian mixture models. Whereas before we made use of a unimodal prior containing all stimulus categories Schoenmakers et al., 2013, here we use Gaussian mixture models where the image prior is taken to be multimodal, by splitting the prior into separate groups of images. Each group results in a mixture component that embodies a set of images from the same category. The weights for the mixture components are estimated from the fMRI data. The BOLD-response is translated to image space and the resemblance to each mixture component is calculated resulting in probabilities of belonging to each category. These probabilities then enforce or suppress the mixture components in the reconstruction. The components can be generated in a supervised way by splitting the prior in separate semantic categories based on image labels (Schoenmakers et al., 2014) or the components can be estimated automatically from the prior by learning cluster assignments based on the stimulus features when categorical information for the prior is unavailable.

Previous studies have shown that it is possible to get an accurate read-out of the category of a perceived image from fMRI data (Haxby et al., 2001; Cox and Savoy, 2003; Kriegeskorte et al., 2008; Simanova et al., 2014). Naselaris et al. (2009) showed that image reconstruction improved when semantic information was incorporated in the forward model. In our framework semantic information from higher order brain areas can be incorporated by deriving semantic categories from high-level brain areas using multinomial logistic regression. This categorical information can then be gated to the prior to enforce the correct categorical information during reconstruction. In some cases it can be difficult to derive the category of handwritten characters from their low-level features, but people are very well adapted to identifying the correct category of ambiguous characters. Hence, by gating higher level brain information ambiguous stimuli might be resolved more accurately.

We applied the Gaussian mixture model to reconstruct multiple handwritten characters that have been presented to subjects during fMRI acquisition using a rapid event-related design. We compare the reconstruction results for supervised multimodal decoding, in which stimulus categories are known, and unsupervised multimodal decoding, in which stimulus categories are unknown, with unimodal decoding which was the method proposed in our previous work (Schoenmakers et al., 2013). Furthermore, we extend the supervised approach to allow for semantic gating, incorporating information from high-level visual areas to drive the prediction of stimulus category. We show a major improvement with more accurate reconstructions than could be obtained using unimodal decoding. The key feature of our approach is the simplicity of our analytical hybrid Bayesian network while not having to make concessions on reconstruction quality.

## 2. Material and methods

### 2.1. Encoding

As in Schoenmakers et al. (2013), we use a linear Gaussian encoding model with image ***x*** = (*x*_1_, …, *x*_*p*_)′ ∈ ℝ^*p*^ and the associated measured brain response ***y*** = (*y*_1_, …, *y*_*q*_)′ ∈ ℝ^*q*^:

(1) $$y=B^{\prime }x+\epsilon$$

with **ϵ** zero-mean normally distributed noise. Regression coefficients ***B*** are estimated using regularized linear regression as in Güçlü and van Gerven (2014) since it is computationally fast. The likelihood function is then given by

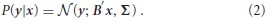



where ***B*** = (**β**_1_, …, **β**_*q*_) ∈ ℝ^*p* × *q*^ and **Σ** = diag(σ^2^_1_, …, σ^2^_*q*_) ∈ ℝ^*q* × *q*^. We assume that this mapping is independent of the context (e.g., the category). Let ***X*** = (***x***^1^, …, ***x***^*N*^)′ ∈ ℝ^*N* × *p*^ denote the design matrix where ***x***^*j*^ denotes the stimulus presented at the *j*-th trial. Let ***y***_*i*_ = (*y*^1^_*i*_, …, *y*^*N*^_*i*_) denote the associated responses for the *i*-th voxel. For each voxel *i*, we minimize the *l*_2_-penalized least squares loss function to estimate **β**_*i*_:

(3) $${\hat{\beta }}_{i}=\underset{\beta_{i}}{\text{arg min}}\;\left[\frac{1}{N}||y_{i}-X\beta_{i}|{|}_{2}^{2}+\lambda_{i}||\beta_{i}|{|}_{2}^{2}\right]$$

where λ_*i*_ ≥ 0 controls the amount of regularization. Coefficients $\hat{\beta }$_*i*_ can be obtained as follows:

(4) $${\hat{\beta }}_{i}={\left(X^{\prime }X+\lambda_{i}I_{p}\right)}^{-1}X^{\prime }y_{i}.$$

For efficiency we can use a singular value decomposition to reduce the complexity of the estimation of **β**_*i*_ from 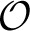
(*p*^3^) to 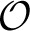
(*pN*^2^) (Hastie et al., 2009; Murphy, 2012).

We use stratified *K*-fold cross-validation with *K* = 5 to estimate λ_*i*_ and σ^2^_*i*_. We first define a grid of values **Λ**_*i*_ = (λ^1^_*i*_, …, λ^*L*^_*i*_) based on the effective degrees of freedom (Güçlü and van Gerven, 2014). Next, we obtain $\hat{\lambda }$_*i*_ as:

(5) $${\hat{\lambda }}_{i}=\underset{\lambda \in \Lambda }{\text{arg min}}\left\{var\left({\hat{\epsilon }}_{i}^{1}{\left(\lambda \right)}^{\prime },\ldots ,{\hat{\epsilon }}_{i}^{K}{\left(\lambda \right)}^{\prime }\right)\right\}$$

where ${\hat{\epsilon }}_{i}^{k}(\lambda )=y_{i}^{k}-X^{k}{\hat{\beta }}_{i}$ are the residuals that are estimated using regularization parameter λ in the *k*-th cross-validation fold with superscript *k* restricted to the trials belonging to that fold. Finally, we obtain $\hat{\sigma }$^2^_*i*_ as:

(6) $${\hat{\sigma }}_{i}^{2}=var\left({\hat{\epsilon }}_{i}^{1}{\left({\hat{\lambda }}_{i}\right)}^{\prime },\ldots ,{\hat{\epsilon }}_{i}^{K}{\left({\hat{\lambda }}_{i}\right)}^{\prime }\right).$$

For the purpose of decoding, only the most informative voxels with $\hat{\sigma }$^2^_*i*_ ≤ 0.99 are included in the model.

### 2.2. Decoding

In our probabilistic framework, decoding comes down to computing the probability of a reconstruction ***x*** given an fMRI response vector ***y*** and cluster assignment *c*. Following standard probabilistic inference, see e.g., Bishop (2006), we obtain

(7) $$P(x|y)=\sum_{c}P(x|y,c)P(c|y)\;,$$

where both *P*(***x***|***y***, *c*) and *P*(*c*|***y***) follow from the application of Bayes' rule:

(8) $$P(x|y,c)=\frac{P(y|x)P(x|c)}{P(y|c)}\text{ and }P(c|y)=\frac{P(c)P(y|c)}{\sum_{c}P(c)P(y|c)}\;,$$

with

(9) P(y|c)=∫dx P(y|x)P(x|c) .

Since both the likelihood *P*(***y***|***x***) and the prior *P*(***x***|*c*) have the form of a Gaussian in ***x***, so does their product. The derivation of (8) and (9) can be found in the supplementary. Here we merely state the result.

The posterior *P*(***x***|***y***, *c*) of a reconstruction ***x*** given the brain response ***y*** under the assumption that the corresponding cluster equals *c* is a Gaussian distribution with mean ***n***_*c*_(***y***) and variance ***Q***_*c*_, which can be computed through

(10) $$n_{c}(y)=Q_{c}\bar{f}(y)+U_{c}m_{c}\;,$$

where

(11) $$\begin{array}{l} U_{c}\equiv {\left(I+R_{c}D\right)}^{-1},\;D\equiv B\Sigma^{-1}B^{\prime },\;Q_{c}\equiv U_{c}R_{c}\text{ , and }\\ \;\bar{f}(y)\equiv B\Sigma^{-1}y\;, \end{array}$$

and with ***I*** the identity matrix. The posterior probability *P*(*c*|***y***) of cluster *c* given the brain response ***y*** can be shown to obey

(12) $$\begin{array}{l} \log P(c|y)=\log\pi_{c}+\frac{1}{2}\log\text{ det}U_{c}+\frac{1}{2}\bar{f}{\left(y\right)}^{\prime }Q_{c}\bar{f}(y)\\ \;-\frac{1}{2}m_{c^{\prime }}DU_{c}m_{c}+\bar{f}{\left(y\right)}^{\prime }U_{c}m_{c}+\text{constants }, \end{array}$$

where the constants can be ignored since they are independent of ***c*** and drop out when normalizing *P*(***c***|***y***) to sum to one. For the final reconstruction we propose to consider

(13) $$x^{*}(y)=\sum_{c}w_{c}(y)n_{c}(y)\text{ with weights }w_{c}(y)\propto P{\left(c|y\right)}^{1/T}\;.$$

For temperature *T* = 1, we have *w*_*c*_(***y***) = *P*(*c*|***y***) and the reconstruction is a standard weighted average of the reconstructions for each of the clusters. In the limit *T* ↓ 0, we zoom in on the reconstruction ***n***_c^*^_(***y***) corresponding to the most probable cluster c^*^ = argmax_*c*_ *P*(*c*|***y***). In previous work we found that the best reconstructions are obtained as *T* ↓ 0 (Schoenmakers et al., 2014), so here only results are reported for the most probable cluster c^*^.

### 2.3. Specification of the image prior

In previous work (Schoenmakers et al., 2013) we used a unimodal prior which contained the images of all categories in one prior distribution over images. In the proposed mixture model we consider the prior *P*(***x***) to consist of multiple Gaussian mixture components. That is,

(14) $$P(x)=\sum_{c}P(c)P(x|c)$$

with *P*(*c*) the prior probability of cluster *c* and *P*(***x***|*c*) = 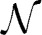
(***x***; ***m***_*c*_, ***R***_*c*_) the Gaussian distribution of cluster *c* with mean ***m***_*c*_ and covariance ***R***_*c*_. All images in the prior are normalized whereafter per cluster the mean 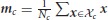 and covariance 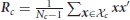 are calculated, with 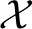
_*c*_ the set of all images that belong to category *c* and *N*_*c*_ = |
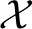
_*c*_|.

For the prior we use a separate set of images taken from the same database as the stimuli used for the fMRI experiment (van der Maaten, 2009). The prior set includes 700 unique instances per character, giving a total of 4200 handwritten characters. The Gaussian mixture components are realized in two ways. When the image categories are known, the components can be split in a supervised fashion leading to supervised multimodal decoding. In case the image categories are not known, a set of images can be split in clusters in an unsupervised way based on how similar features are between images, resulting in unsupervised multimodal decoding. For supervised multimodal decoding the prior is subdivided in six semantic categories (B, R, A, I, N, and S) as they are categorized in the database of handwritten characters. For unsupervised multimodal decoding the prior is divided into a variable number of clusters as obtained by K-means clustering. The K-means algorithm groups together characters that have similar low-level features (Spath, 1985; Seber, 2009). To obtain the Gaussian for cluster *c*, we take the cluster mean ***m***_*c*_ provided by the K-means algorithm and calculate the covariance ***R***_*c*_ as stated above.

### 2.4. Semantic gating

By default *P*(*c*) can be specified as a uniform probability over categories, so as 1/*C*. Alternatively, we can model *P*(*c*|***z***) by incorporating semantic information derived from higher-order brain areas. We propose to learn these probabilities with multinomial logistic regression leading to a mechanism reminiscent of the mixture-of-experts by Jordan and Jacobs (1994). We will refer to this approach as semantic gating.

Assume we have a stimulus-response pair in the form of a set of clusters *c* ∈ (1, …, *C*) and associated measured brain response ***z*** = (*z*_1_, …, *z*_*q*_)′ ∈ ℝ^*q*^ where ***z*** refers to the brain responses of a higher-level brain area. According to the multinomial logistic regression model, the probability of the category given the brain responses is given by:

(15) $$P\left(c|z\right)=\frac{\exp\left(\alpha_{c}+{\gamma^{\prime }}_{c}z\right)}{\sum_{k=1}^{K}\exp\left(\alpha_{k}+{\gamma^{\prime }}_{k}z\right)}.$$

We take the approach in Friedman et al. (2010) to estimate the regression coefficients. We maximize the *l*_1_-penalized log-likelihood to estimate {α_*k*_, **γ**_*k*_}^*K*^_*k* = 1_ and use 10-fold cross-validation on the train set to estimate the λ that controls the amount of regularization:

(16) $$\begin{array}{l} {\left\{{\hat{\alpha }}_{k},{\hat{\gamma }}_{k}\right\}}_{k=1}^{K}=\underset{{}_{{\left\{\alpha_{c},\gamma_{c}\right\}}_{c=1}^{K}}}{\text{arg max}}\{\frac{1}{N}\sum_{j=1}^{N}\log P\left(c^{i}|z^{i}\right)\\ \;-\lambda \sum_{k=1}^{K}||\gamma_{k}|{|}_{1}\}. \end{array}$$

**Figure 1:**
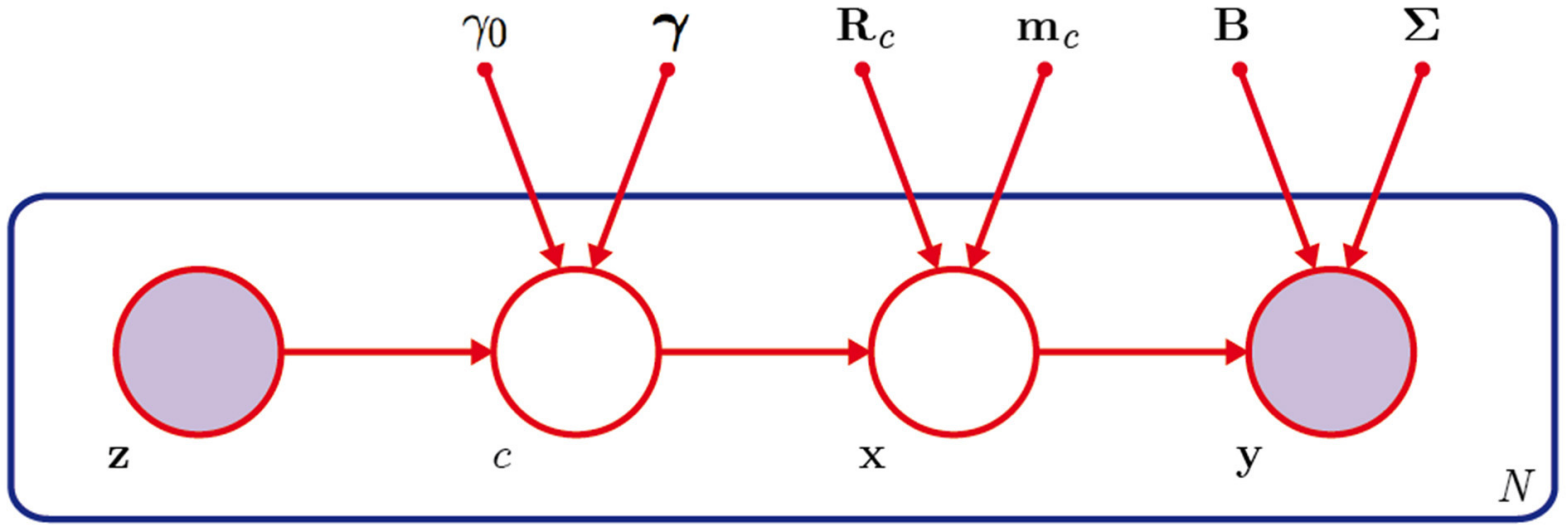
**The hybrid Bayesian network**. The probability of belonging to cluster *c* is estimated from the brain response ***z*** from a high-level brain area with the help of γ_0_ and **γ**. An encoding model provides regression coefficients ***B*** and covariance **Σ** to reconstruct the image from the brain response ***y*** of a lower-level brain area. The mean ***m***_*c*_ and covariance ***R***_*c*_ of a set of prior images enforce structure and the cluster *c* enforces semantic information during the reconstruction of image ***x***. This process can be repeated for a set of *N* images.

Our model is visualized in Figure 1, showing a graphical representation of the Gaussian mixture model with semantic gating and the variables that guide the reconstruction of a handwritten character from the brain response.

### 2.5. fMRI experiment

#### 2.5.1. Participants

In this study three healthy native Dutch speaking participants have been recruited to view stimuli of handwritten characters. All participants gave written consent according to the institutional guidelines set forth by the local ethics committee (CMO region Arnhem Nijmegen, the Netherlands) before the experiment. The participants were not paid for participation.

#### 2.5.2. Stimuli

The stimuli represented grayscale handwritten characters centrally presented on a black background. The images spanned 9 × 9 degrees of visual angle (56 × 56 pixels). All character images used in this study came from a database that was previously collected by van der Maaten (2009). The character database consists of 40,000 handwritten characters collected from 250 writers. The images in the database are rescaled and centered such that they fill the canvas. For this study six characters were selected: B, R, A, I, N, and S. For each character, 60 unique instances were presented during the experiment. A total of 360 characters were shown and this was repeated once in order to get a better estimate of the BOLD response (see FMRI data preprocessing section). The images were shown as flickering stimuli (200 ms ON, 200 ms OFF) for the duration of 1 sec, followed by 3 sec of black background. A central white square served as a fixation point (0.2° of visual angle). The fixation point was present at the center of the screen throughout the whole experiment.

#### 2.5.3. Procedure

To keep participants vigilant they were asked to focus on the fixation point and to respond with a button press when the fixation point changed color from dark gray to light blue. The fixation point changed color once every six stimuli on average. Changes were presented at random but evenly spread over the length of the experiment and counterbalanced over characters. The characters were shown in pseudo-random order by shuffling six character sets consisting of one instance of each character in order to prevent long repetitions of the same character. The experiment lasted for 50 min with a self-paced rest period in the middle. After the experiment, a structural scan was made. Subsequently, or in a next session, participants viewed a rotating checkerboard wedge in order to localize the visual areas in the brain with polar retinotopy. The rotating wedge was presented in four blocks of 5 min.

#### 2.5.4. fMRI acquisition

Imaging was conducted at the Donders Institute for Brain, Cognition and Behavior (Nijmegen, the Netherlands). The functional images were collected with a Siemens Trio 3 T MRI system (Siemens, Erlangen, Germany) with an EPI sequence using a 32 channel head coil (TR = 1.74 s, TE = 30 ms, GRAPPA acceleration factor 3, 83° flip angle, 30 slices in ascending order, voxel size 2 × 2 × 2 mm). Head movement was restricted with foam cushions and a tight strip of tape over the forehead. After functional imaging, a structural scan was acquired using an MPRAGE sequence (TR = 2.3 s, TE = 3.03 ms, voxel size 1 × 1 × 1 mm, 192 sagittal slices, FoV = 256 mm). In a separate session, the functional localizer data was acquired, again using an EPI sequence (TR = 2 s, TE = 30 ms, 83° flip angle, 33 slices in ascending order, voxel size 2 × 2 × 2 mm, FoV = 192 mm). During acquisition an eye tracker was employed to verify if participants were fixating their gaze.

#### 2.5.5. fMRI preprocessing

With the use of SPM8 software (Wellcome Department of Imaging Neuroscience, University College London, UK), the functional volumes were reconstructed, realigned to the first scan of the session and slice time corrected. Participants moved less than 0.5 mm during the sessions. For each unique stimulus, which was presented twice to the subject, the response of each voxel to the stimulus was computed using a general linear model (GLM). The design matrix of the GLM was shaped by one regressor encoding the two stimulus repetitions, one regressor encoding all other stimuli, and nuisance regressors that encoded movement parameters and drift terms, similar to the approach presented in Mumford et al. (2011). The design matrix was convolved with the canonical hemodynamic response function (HRF). The voxel response for each stimulus was given by the beta estimate which was normalized for each voxel. Freesurfer software was used together with functional localizer data in order to isolate voxels belonging to visual area V1 and V2 using established methods for retinotopy (Sereno et al., 1995; DeYoe et al., 1996; Engel et al., 1997).

### 2.6. Empirical validation

In order to validate our approach we estimated an encoding model for the fMRI data from visual area V1 and tested different versions of decoding under the Gaussian mixture model approach. In order to examine whether semantic information can improve decoding, we compared supervised multimodal decoding and unsupervised multimodal decoding with unimodal decoding. In the supervised multimodal setting we also tested whether including semantic information from a higher-level visual area (V2) with semantic gating of the six character categories will help improve reconstructions.

To quantify the reconstruction performance we used two measures. The structural similarity metric (SSIM) was used to see how well low-level image features are reconstructed. SSIM is designed to match the properties of the human visual system when determining to what extent two images are alike (Wang et al., 2004). SSIM is similar to correlation between images, ranging from 0 to 1, except that it controls for noise and distortion and only evaluates the structural congruency between the original images and their reconstructions. Secondly, we computed correct classifications to gain insight into how well high-level semantic information was decoded. In supervised decoding, the letter category *l* coincides with the class *c* and we measure the classification performance by counting the number of correctly classified test set images by assigning images to the most probable class and thus letter category. For unsupervised decoding, we compute the probability *P*(*l*|***y***) by multiplying the probability of each cluster assignment *P*(*c*|***y***) with the probability of each letter category *P*(*l*|*c*) and summing over the clusters *c*. Here we estimate the probability *P*(*l*|*c*) through the frequency distribution over letter categories of the images in the prior image set that are assigned to cluster *c*. The test set image is then again classified as the letter category with the highest probability *P*(*l*|***y***). Note that for unimodal decoding classification performance is not objectively measurable and thus not provided. For unsupervised decoding we repeated the procedure ten times for each number of clusters because K-means finds different clusterings each time it is executed, which leads to variability in reconstruction quality. We used two-tailed *t*-tests to compare mean decoding performances.

Furthermore, as a baseline, we compared our approach with a simple discriminative approach to form reconstructions. This is achieved by directly predicting pixel values from observed fMRI responses using the same ridge regression approach as used in the encoding model. Also, we compared the classification performance of our approach with a simple discriminative approach. This is achieved by directly predicting class labels from observed brain responses using multinomial logistic regression with *l*_1_ penalty. We tested the robustness of our generative model by extending the prior to contain the full English alphabet instead of the subset of the six character categories. The character database does not contain the letter “X” so those were not included. Furthermore, some of the characters did not have 700 exemplars to contribute. These were oversampled to arrive at 700 instances of each category providing for a more balanced analysis.

## 3. Results

**Figure 2:**
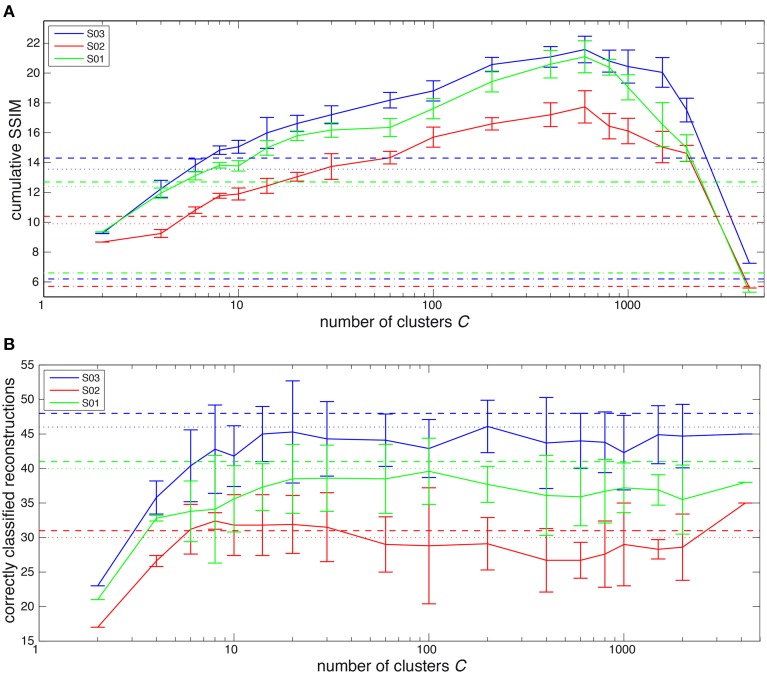
**(A)** Summations over the SSIM score for all original images with their reconstructions. The dashed-dotted lines show the scores for unimodal reconstruction, the dotted lines show the scores for supervised multimodal decoding and the dashed lines show the SSIM scores for supervised multimodal decoding scores with semantic gating. The solid lines indicate the SSIM score for different numbers of clusters for unsupervised multimodal reconstruction. The error bars indicate the standard error of the mean and the colors indicate the different subjects S01, S02, and S03. **(B)** Number of reconstructions that were classified correctly in the test set of 72 images for all participants. The dotted lines show the classification performance for supervised multimodal decoding and the dashed lines show the classification for supervised multimodal decoding with semantic information included. For unsupervised multimodal decoding the mean and range are given for different numbers of clusters.

Figure 2 shows the summation of SSIM scores for the original images in the test set and their reconstructions in Figure 2A. In Figure 2B the number of correctly classified reconstructions are shown for the different forms of decoding. Figure 2A shows that all multimodal forms of decoding perform significantly better than unimodal decoding except for unsupervised decoding with 4200 clusters (*p* < 10^−4^ for unimodal vs supervised multimodal, supervised multimodal with semantic gating and unsupervised multimodal except for *C* = 4200). Semantic gating gives a weakly significant increase over supervised multimodal decoding without semantic gating (*p* = 0.0139 over all participants). Unsupervised multimodal decoding outperforms supervised multimodal decoding when the number of clusters exceeds eight for all participants. When the number of clusters increases the SSIM scores go up until the prior is split up into more than 600 clusters, after which the SSIM scores start to drop again. The classification performance strongly increases when the prior is split up into a small number of classes, but from six clusters onwards the classification performance remains relatively stable. Classification is similar in performance for both supervised and unsupervised decoding, except that unsupervised decoding results are much more variable.

**Figure 3:**
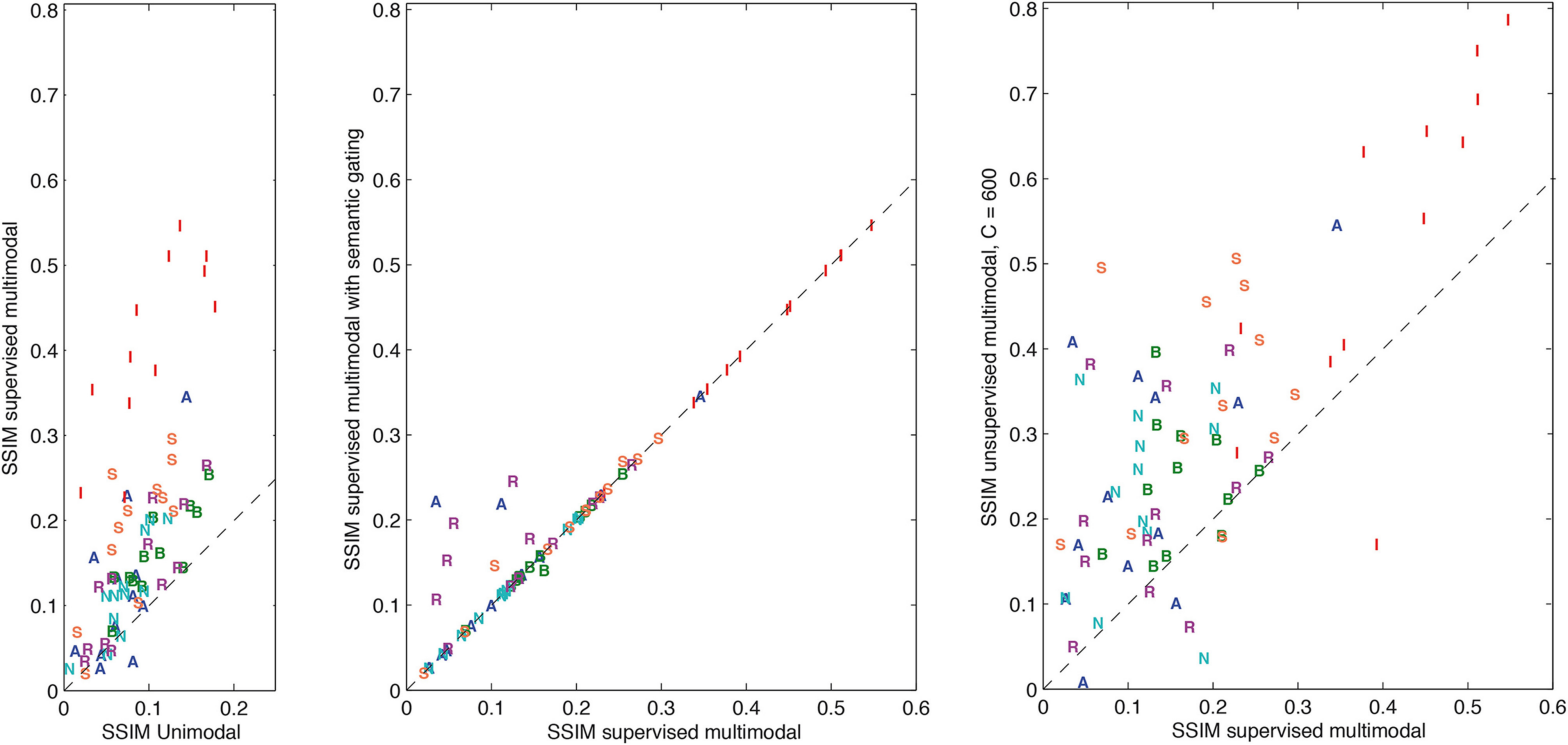
**Comparisons between the different forms of decoding for the individual instances in the test set for S03 measured in terms of SSIM**. First unimodal decoding vs. supervised multimodal decoding, then supervised multimodal decoding with or without semantic gating and finally supervised multimodal decoding vs. unsupervised multimodal decoding.

Figure 3 shows comparisons between the different forms of decoding for the individual instances in the test set for subject S03. In the left-most panel the results for unimodal decoding vs. supervised multimodal decoding are shown. Almost all reconstructions improve for multimodal decoding. The middle panel shows that semantic gating increases the reconstruction accuracy of a small portion of the test set, while for most of the reconstructions the performance stays the same as the performance when reconstructing without semantic information. One instance is reconstructed worse than without semantic information. The right-most panel compares supervised decoding to unsupervised multimodal decoding with 600 clusters. The great majority of instances improves for unsupervised multimodal decoding in comparison with supervised multimodal decoding. A salient finding is that especially the reconstructions of the characters “I” are greatly improved under all forms of multimodal decoding.

**Figure 4:**
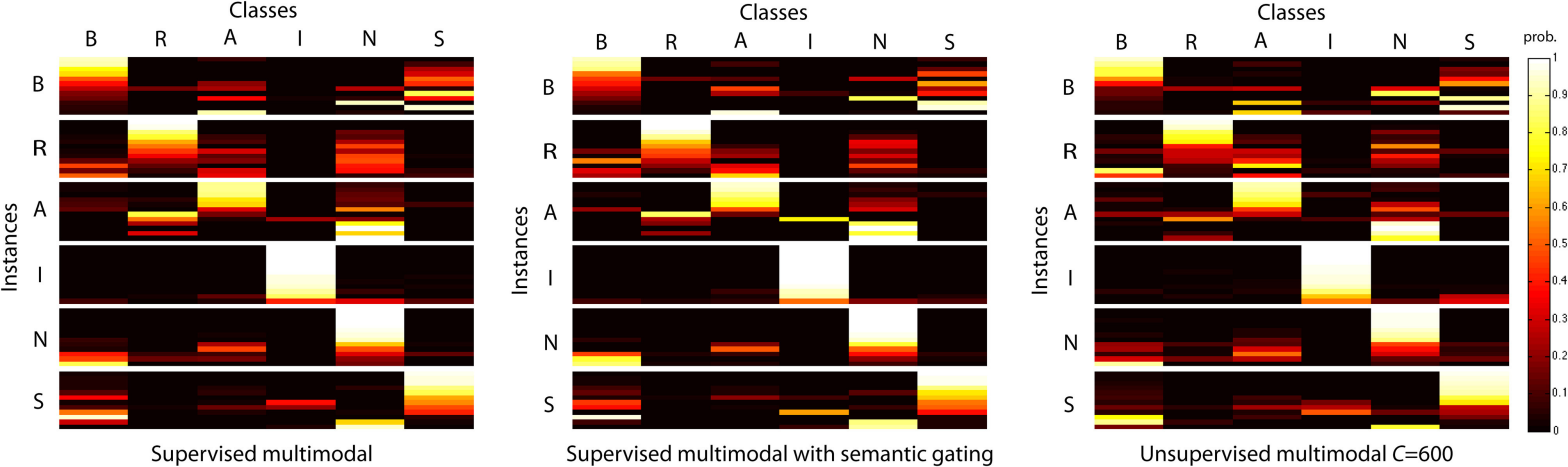
**The probabilities for all instances of the test set on the six character categories for S03 for supervised multimodal decoding, supervised multimodal decoding with semantic gating and unsupervised multimodal decoding**. The instances have been sorted on the probability of the correct category.

Figure 4 depicts the probability of belonging to each category for all instances in the test set for subject S03. The classification performance is similar for both supervised and unsupervised decoding. The block diagonal structure demonstrates that many of the instances are correctly identified with a high probability for both supervised and unsupervised decoding. Furthermore, the figure reveals that often one or just a few categories are attributed to an instance. Often the most probable category is the category to which the instance belongs. Semantic gating increases accuracy for some instances and decreases accuracy for other instances. Furthermore, the figures reveal that some characters are confused with each other. For instance, characters “A” and “N” are often confused and also the character “B” is confused with all other categories. The character “I” is an example of a character that is not often confused with the other categories.

**Figure 5:**
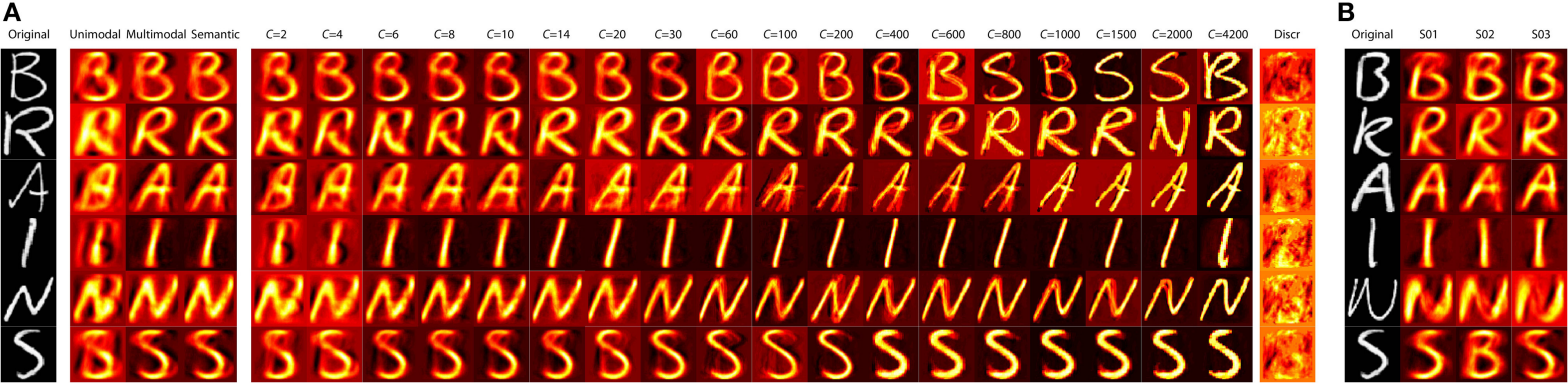
**(A)** shows exemplars of reconstructions for S03 for the different decoding variants. **(B)** shows exemplars of original images and their reconstructions for the different participants using supervised multimodal decoding.

Figure 5 displays the reconstruction results for the different decoding schemes for some exemplars in the test set for subject S03. As can be seen, unimodal decoding retrieves the gist of the original stimulus but multimodal decoding improves the reconstructions greatly. Furthermore, it is shown that supervised multimodal decoding and unsupervised multimodal decoding with more than six clusters results in reconstructions of similar quality. For unsupervised multimodal decoding with an increasing number of clusters the reconstructions are sharpened. When the number of clusters exceeds about 20 the reconstructions start to converge to a particular image from the prior, which might not fully fit the original, but has a very high overlap. When the number of clusters stays under approximately 20, the reconstruction is a mix of a large number of prior images. Below approximately 20 clusters the reconstructions match the original very well, but the reconstructions are more blurry than the original. Figure 5B shows some exemplars that convey that the reconstructions are very similar across participants. Since we choose to take the most probable cluster for reconstruction we obtain high-quality image reconstructions, but in cases where a cluster is chosen that represents the incorrect category, the reconstructions are similar in their low-level features, yet incongruent in terms of their semantics. For instance in Figure 5B the “S” for S02 is reconstructed as a “B” for which the low-level features match the original quite well.

As a baseline, we test a basic discriminative model with the same ridge regression approach. The summation of SSIM scores in the test set result in 1.44, 0.29, and 0.41 for subjects S01, S02, and S03 respectively, which is significantly lower than all decoding settings including unimodal decoding (unimodal: *p* = 0.001, other: *p* < 10^−4^ over all participants). For comparison, learning the categories in a discriminative setting from V1 with *l*_1_-penalized multinomial logistic regression resulted in 54.2, 44.4, and 51.4% correctly classified test set images for S01, S02, and S03, respectively. This is not significantly different from the results for multimodal decoding with 55.6, 41.7, and 63.9% for each of the participants. Similarly there is no significant difference for supervised multimodal decoding with semantic gating or unsupervised multimodal decoding.

**Figure 6:**
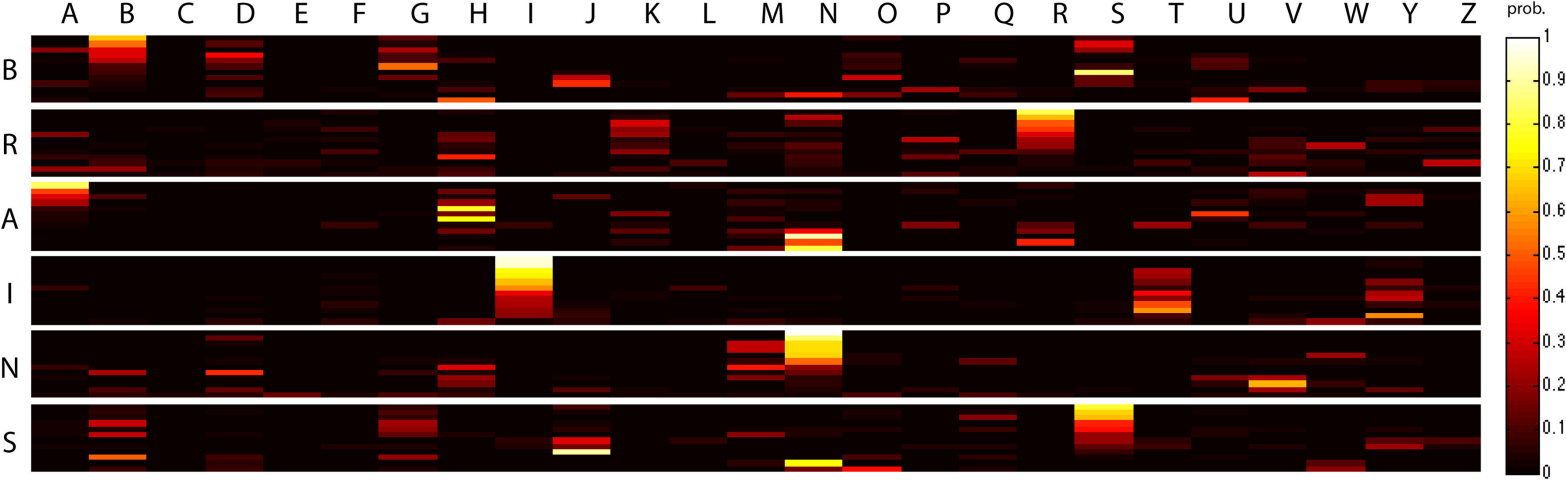
**Probability for all 72 instances of the test set for S03 with the extended prior that contains instances for the characters of the alphabet**. The instances have been sorted on the probability of the correct category.

Figure 6 illustrates the probabilities of belonging to the character categories for all test set images when the prior is extended to include the complete alphabet. Still a significant number of images is correctly identified. We obtained accuracies of 30.6, 16.7, and 45% over a 4% chance level for each of the three participants. It is remarkable how well the characters still converge to the correct category for subject S03. However, for the participants' data that give rise to less accurate reconstructions, characters often become assigned to the incorrect categories. For S03 it can be observed that only a few of the other categories are selected and that these categories are often very similar in their low-level features with the correct category. Previously, the “I” was never confused with the other categories, while now the “T” becomes a confusing category. This shows that characters are easy to classify when they are unique in their low-level features, which is particularly the case for the character “I” in our dataset. In contrast, Figure 6 shows that the character “A” is a very difficult character to reconstruct, possibly because of strongly overlapping features with other characters, such as the “H” and the “N”. The reconstruction performance in terms of SSIM also slightly decreases to 9.3, 8.4, and 12.3 for S01, S02, and S03, respectively, using supervised multimodal decoding given the extended prior.

## 4. Discussion

We introduced a hybrid Bayesian network that can decode stimuli from the human brain by considering both low-level feature information and high-level semantic information. In the network, information from low-level brain areas comes together with information from high-level brain areas when using semantic gating. Results show that the reconstruction performance is very accurate for the majority of stimuli and highly improved in contrast to decoding in a unimodal setting. Furthermore, we showed that multimodal decoding can be fully automated by learning clusters from the data, leading to equally good decoding performance.

While discriminative models work well, it has been shown that generative models in many cases perform better in settings with few training samples (Ng and Jordan, 2002). Often fMRI datasets are recorded under tight constraints on scanning time and attention span from participants. Our generative approach makes accurate reconstructions from fMRI data feasible under such circumstances. Our data is recorded under a standard approach of a rapid event-related design with an hour of scanning, which is not too demanding for participants. Using a relatively small set of prior images in the Gaussian mixture model we could obtain accurate reconstructions. A simple regularized linear regression under the same settings with a discriminative approach for reconstruction of images at pixel level results in very poor reconstructions contrary to our generative approach. With some effort, similar reconstruction performance can probably be obtained with more advanced discriminative or generative approaches, in particular those that implicitly or explicitly involve dimension reduction (e.g., Miyawaki et al., 2008; van Gerven and Heskes, 2010; van Gerven et al., 2010). However, such models may be more difficult to interpret and to extend than the generative Bayesian framework advocated here.

A strength of our generative approach with a multimodal prior is that we can automatically infer higher-order semantic categories from low-level image features. We found that biasing toward the most probable category leads to better reconstructions (in terms of SSIM) than taking a standard weighted average over all categories. The drawback of choosing the most probable category is that reconstructions may converge toward the incorrect stimulus category. It is not always possible to correctly classify handwritten characters based on their low-level features. Sometimes the characters are ambiguous about their category and often the category with which it is confused is actually a category that has highly overlapping features. Furthermore, post hoc exploration of the prior revealed that approximately ten of the original images of the test set are very similar to instances from the prior that come from a different category. So even if the BOLD response would have been recorded perfectly for these instances we would end up with wrongly categorized reconstructions. This suggests that perfect classification is impossible based on low-level features alone, which might be avoided when high-level brain areas can contribute the correct classification for categorically ambiguous images.

In order to overcome the incorrect classification of stimuli based on their low-level information, semantic gating can improve the classification and thus the reconstruction. By extracting semantic information from higher-order brain areas we were able to enforce the correct category during reconstruction. Here, we only gated information from V2 since we did not have coverage of other high-level brain regions for this dataset. The dataset was initially gathered to get the best possible acquisition of V1 and therefore extrastriate cortex was not fully available. It is quite impressive that including categorical information from V2 alone already gives an improvement, since V2 is still a relatively low-level visual area. Future work can investigate if there is a greater improvement when categorical information from the complete visual hierarchy is included. When an image is shown it will propagate through the brain, so each visual area should boost the reconstructions and classification performance. Furthermore, studies with stimuli from a wide range of semantic categories could greatly benefit from this paradigm. The semantic space in the brain can be mapped like in Huth et al. (2012). This semantic information can then be gated in order to tailor the prior to the stimulus that is decoded from V1, possibly resulting in scaling up the image reconstruction to a wide range of categories.

In this experiment, we had access to a fully labeled dataset which made it easy to explore a supervised model, but a fully labeled database might not be available in all settings. Especially when we want to extend the reconstruction paradigm to the full range of natural images an extremely large set of images is probably necessary to span the space of natural images, in which case labels are probably not available. Therefore, we investigated whether the reconstructions can be made with a prior for which the mixture components were estimated in an unsupervised setting. We showed that it is possible to have a fully automated setup that infers a fitting set of images from the prior based on low-level information without sacrificing reconstruction performance. In our case the optimum reconstruction seems to be reached when the set of images in the prior is subdivided in 600 clusters. This optimum might be different for each dataset, but can be learned from the data. In principle, it is also possible to apply semantic gating to unsupervised decoding, but in our case this did not give good results (investigated, but not reported in this paper). Our dataset contained 360 recordings of stimuli, which is enough data for six categories when the dataset is well balanced. Unfortunately the K-means cluster assignments rarely result in a well-balanced split. With only few stimuli it is hard to get a good estimation with multinomial logistic regression, resulting in poor decoding performance.

It is difficult to find a good measure for comparing images. Here, we used a combination of two types of measurements, structural similarity metric and classification performance. Both measures cover different aspects of the tested images and therefore give different results for image comparison. Together they allow for objective comparison, but still not all reconstruction information is encompassed by these measures. An alternative albeit time consuming way to evaluate reconstruction performance could be to acquire subjective ratings with a behavioral experiment.

We showed that high-quality reconstructions can be obtained from human brain data, but our framework can be advanced further. Empirically, we observed that it is important to have a good voxel selection to get the framework to perform sufficiently well. The voxel selections we made were based on retinotopic mapping and selecting the voxels with high explained variance. This selection may be improved further by explicitly modeling sparseness in the voxel domain during encoding. Moreover, we here used a canonical HRF but the shape of the HRF varies across brain regions and subjects (Handwerker et al., 2004; Badillo et al., 2013). If the HRF is tailored to individual voxels, the performance is expected to increase for both encoding and decoding (Pedregosa et al., 2013). Another improvement could be to make use of a richer prior. In our dataset not all original images were accurately represented by the prior. A multi-scale approach might make it possible to include decoding of under-represented mid-level image features independent of semantic category.

An interesting avenue for future research is to examine how our framework performs on more challenging datasets while using more brain regions to drive the reconstructions. Also, it would be interesting to examine the merits of our framework when reconstructing the contents of other sensory modalities, of subjective states such as mental imagery or internal speech, or to reconstruct motor output.

In summary, we have developed a hybrid Bayesian network that can combine different sources and levels of information in a natural way to yield accurate reconstructions of handwritten characters from brain responses.

### 4.1. Data sharing

The data and code can be obtained through our lab website: www.ccnlab.net. Please refer to this article when using our data or code.

## Author contributions

Sanne Schoenmakers, Marcel van Gerven, and Tom Heskes designed the experiments. Sanne Schoenmakers, Umut Güçlü, Marcel van Gerven, and Tom Heskes developed the methods. Sanne Schoenmakers collected the data and carried out the analysis. Sanne Schoenmakers, Umut Güçlü, Marcel van Gerven, and Tom Heskes prepared the manuscript.

### Conflict of interest statement

The authors declare that the research was conducted in the absence of any commercial or financial relationships that could be construed as a potential conflict of interest.
