# Supplementary material for: Gaussian mixture models and semantic gating improve reconstructions from human brain activity
Source: Front Comput Neurosci. 2015 Jan 30;8:173. doi: 10.3389/fncom.2014.00173 (PMC4311641; doi:10.3389/fncom.2014.00173)
Supplement: Supplementary file 1 [file DataSheet1.PDF]

Frontiers in Neuroscience

Research Article
19 January 2015

Gaussian mixture models and semantic gating improve reconstructions from human brain activity

Sanne Schoenmakers [1,*], Umut Güçlü [1], Marcel van Gerven [1] and Tom Heskes [2]

[1] *Radboud University Nijmegen, Donders Institute for Brain, Cognition and Behaviour, The Netherlands*
[2] *Radboud University Nijmegen, Institute for Computing and Information Sciences, The Netherlands*

Correspondence*:
Sanne Schoenmakers
Radboud University Nijmegen, Donders Institute for Brain, Cognition and Behaviour, Montessorilaan 3, P.O. Box 9104, 6500 HE, Nijmegen, The Netherlands, www.ccnlab.net, s.schoenmakers@donders.ru.nl

Bayesian networks in neuroscience

1 SUPPLEMENTAL DATA

Here, we derive the analytical expression for the posterior $P(\boldsymbol{x}|\boldsymbol{y}, c)$ of a reconstruction $\boldsymbol{x}$ given the brain response $\boldsymbol{y}$ for cluster c and the posterior probability $P(c|\boldsymbol{y})$ of cluster c given the brain response $\boldsymbol{y}$.

1.1 INFERENCE

The goal is now to compute $P(\boldsymbol{x}|\boldsymbol{y}) = \sum_c P(c|\boldsymbol{y})P(\boldsymbol{x}|\boldsymbol{y}, c)$. Using Bayes' rule we have

$$P(\boldsymbol{x}|\boldsymbol{y}, c) = \frac{P(\boldsymbol{y}|\boldsymbol{x})P(\boldsymbol{x}|c)}{P(\boldsymbol{y}|c)} , \tag{1}$$

with

$$P(\boldsymbol{y}|c) = \int d\boldsymbol{x} \; P(\boldsymbol{y}|\boldsymbol{x})P(\boldsymbol{x}|c) . \tag{2}$$

The posterior over categories then reads

$$P(c|\boldsymbol{y}) = \frac{P(\boldsymbol{y}|c)P(c)}{\sum_{c'} P(\boldsymbol{y}|c')P(c')} . \tag{3}$$

In the following, it is useful to realize that we do not have to keep track of proportionality constants that are independent of $\boldsymbol{x}$ and c: they drop out anyway when we normalize in (1) or (3). Now, we can, up to those irrelevant proportionality constants, turn a multivariate Gaussian on $\boldsymbol{y}$ centered around a linear

function of $\boldsymbol{x}$ into a multivariate Gaussian on $\boldsymbol{x}$ centered around a linear function of $\boldsymbol{y}$:

$$P(\boldsymbol{y}|\boldsymbol{x}) = \mathcal{N}(\boldsymbol{y}; \boldsymbol{B}^{'}\boldsymbol{x}, \Sigma) \propto \mathcal{N}(\boldsymbol{x}; \bar{\boldsymbol{x}}(\boldsymbol{y}), \boldsymbol{\Psi}) \,,$$

with

$$\bar{\boldsymbol{x}}(\boldsymbol{y}) \equiv \left(\boldsymbol{B}\boldsymbol{\Sigma}^{-1}\boldsymbol{B}^{'}\right)^{-1}\boldsymbol{B}\boldsymbol{\Sigma}^{-1}\boldsymbol{y} \text{ and } \boldsymbol{\Psi} \equiv \left(\boldsymbol{B}\boldsymbol{\Sigma}^{-1}\boldsymbol{B}^{'}\right)^{-1} \,.$$

The mean $\bar{\boldsymbol{x}}(\boldsymbol{y})$ is the most likely reconstruction that follows from the Moore-Penrose pseudoinverse: the optimal reconstruction ignoring any prior information, i.e., in the limit of an infinitely flat prior.

Now, the numerator in (1) and hence the integrand in the evidence (2) is (proportional to) a product of two Gaussians:

$$P(\boldsymbol{y}|\boldsymbol{x})P(\boldsymbol{x}|c) \propto \mathcal{N}(\boldsymbol{x}; \bar{\boldsymbol{x}}(\boldsymbol{y}), \boldsymbol{\Psi})\,\mathcal{N}(\boldsymbol{x}; \boldsymbol{m}_c, \boldsymbol{R}_c) \,,$$

where the first Gaussian $P(\boldsymbol{y}|\boldsymbol{x})$ contains the information from the likelihood and the second Gaussian $P(\boldsymbol{x}|c)$ the information from the prior. A product of two multivariate Gaussians is proportional to another multivariate Gaussian. The posterior precision matrix, here denoted $\boldsymbol{Q}_c^{-1}$, follows by adding the precision matrices of the two sources and the posterior mean, here called $\boldsymbol{n}_c(\boldsymbol{y})$, is a weighted combination of the means of the two sources. Here we cannot completely ignore the proportionality constant since it depends on c. Luckily we can make use of a standard result from probability calculus (see e.g., **Petersen and Pedersen** (2008), Section 8.1.8):

$$\mathcal{N}(\boldsymbol{x}; \bar{\boldsymbol{x}}(\boldsymbol{y}), \boldsymbol{\Psi})\,\mathcal{N}(\boldsymbol{x}; \boldsymbol{m}_c, \boldsymbol{R}_c) = \mathcal{N}(\bar{\boldsymbol{x}}(\boldsymbol{y}); \boldsymbol{m}_c, \boldsymbol{\Psi} + \boldsymbol{R}_c)\,\mathcal{N}(\boldsymbol{x}; \boldsymbol{n}_c(\boldsymbol{y}), \boldsymbol{Q}_c) \,,$$

with

$$\boldsymbol{Q}_c \equiv \left(\boldsymbol{\Psi}^{-1} + \boldsymbol{R}_c^{-1}\right)^{-1} \equiv \boldsymbol{\Psi}\left(\boldsymbol{\Psi} + \boldsymbol{R}_c\right)^{-1}\boldsymbol{R}_c = \boldsymbol{R}_c\left(\boldsymbol{\Psi} + \boldsymbol{R}_c\right)^{-1}\boldsymbol{\Psi} \,,$$

and

$$\boldsymbol{n}_c(\boldsymbol{y}) \equiv \left(\boldsymbol{\Psi}^{-1} + \boldsymbol{R}_c^{-1}\right)^{-1}\left(\boldsymbol{\Psi}^{-1}\bar{\boldsymbol{x}}(\boldsymbol{y}) + \boldsymbol{R}_c^{-1}\boldsymbol{m}_c\right) = \boldsymbol{R}_c\left(\boldsymbol{\Psi} + \boldsymbol{R}_c\right)^{-1}\bar{\boldsymbol{x}}(\boldsymbol{y}) + \boldsymbol{\Psi}\left(\boldsymbol{\Psi} + \boldsymbol{R}_c\right)^{-1}\boldsymbol{m}_c \,.$$

Plugging the above into (2), we obtain

$$P(\boldsymbol{y}|c) \propto \mathcal{N}(\bar{\boldsymbol{x}}(\boldsymbol{y}); \boldsymbol{m}_c, \boldsymbol{\Psi} + \boldsymbol{R}_c) \,,$$

or, equivalently,

$$P(\boldsymbol{y}|c) \propto \mathcal{N}(\boldsymbol{m}_c; \bar{\boldsymbol{x}}(\boldsymbol{y}), \boldsymbol{\Psi} + \boldsymbol{R}_c) \,.$$

Roughly speaking, the better the maximum likelihood reconstruction $\bar{\boldsymbol{x}}(\boldsymbol{y})$ matches the prior mean $\boldsymbol{m}_c$ of category c (or vice versa), the higher the likelihood that the voxel activations are generated by an image in this category. The posterior reads

$$P(c|\boldsymbol{y}) \propto \pi_c\,\mathcal{N}(\bar{\boldsymbol{x}}(\boldsymbol{y}); \boldsymbol{m}_c, \boldsymbol{\Psi} + \boldsymbol{R}_c) \,,$$

and should be normalized such that $\sum_c P(c|\boldsymbol{y}) = 1$.

1.2 NUMERICALLY STABLE IMPLEMENTATION

The above may well be mathematically correct, but implementation is doomed to fail when some of the matrices are singular. In our case, the number of voxels is smaller than the number of pixels, which makes that

$$\boldsymbol{D} \equiv \boldsymbol{B}\boldsymbol{\Sigma}^{-1}\boldsymbol{B}^{'} \,,$$

is singular and cannot be inverted to yield $\boldsymbol{\Psi}$. Furthermore, since the number of examples is smaller than the number of pixels, $\boldsymbol{R}_c$ is singular and hence cannot be inverted either.

To prevent inversion of singular matrices, we rewrite

$$\boldsymbol{Q}_c = \left(\boldsymbol{D} + \boldsymbol{R}_c^{-1}\right)^{-1} = \left(\boldsymbol{1} + \boldsymbol{R}_c\boldsymbol{D}\right)^{-1}\boldsymbol{R}_c \equiv \boldsymbol{U}_c\boldsymbol{R}_c \;\text{ with }\; \boldsymbol{U}_c \equiv \left(\boldsymbol{1} + \boldsymbol{R}_c\boldsymbol{D}\right)^{-1} \;.$$

Similarly,

$$\boldsymbol{\Theta}_c \equiv \left(\boldsymbol{D}^{-1} + \boldsymbol{R}_c^{-1}\right)^{-1} = \boldsymbol{D}\boldsymbol{U}_c \,.$$

$\boldsymbol{Q}_c$ and $\boldsymbol{\Theta}_c$ should be symmetric (and may have to be symmetrized); $\boldsymbol{U}_c$ does not have to be symmetric. Since we cannot stably compute $\bar{\boldsymbol{x}}(\boldsymbol{y})$, we instead define

$$\bar{\boldsymbol{f}}(\boldsymbol{y}) \equiv \boldsymbol{\Psi}^{-1}\bar{\boldsymbol{x}}(\boldsymbol{y}) = \boldsymbol{B}\boldsymbol{\Sigma}^{-1}\boldsymbol{y} \,,$$

so that the reconstruction for category i can be computed through

$$\boldsymbol{n}_c(\boldsymbol{y}) = \boldsymbol{Q}_c\bar{\boldsymbol{f}}(\boldsymbol{y}) + \boldsymbol{U}_c\boldsymbol{m}_c \,.$$

The expression for the posterior can be rewritten into

$$\log P(c|\boldsymbol{y}) \;=\; \log \pi_c + \frac{1}{2}\log\det\boldsymbol{U}_c - \frac{1}{2}\left(\bar{\boldsymbol{f}}(\boldsymbol{y}) - \boldsymbol{D}\boldsymbol{m}_c\right)'\left(\boldsymbol{D} + \boldsymbol{D}\boldsymbol{R}_c\boldsymbol{D}\right)^{-1}\left(\bar{\boldsymbol{f}}(\boldsymbol{y}) - \boldsymbol{D}\boldsymbol{m}_c\right) + \text{ constants} \,,$$

we can make use of Woodbury's inversion formula to obtain

$$\left(\boldsymbol{D} + \boldsymbol{D}\boldsymbol{R}_c\boldsymbol{D}'\right)^{-1} = \boldsymbol{D}^{-1} - \left(\boldsymbol{D} + \boldsymbol{R}_c^{-1}\right)^{-1} = \boldsymbol{D}^{-1} - \boldsymbol{Q}_c \,,$$

which then gives

$$-\frac{1}{2}\bar{\boldsymbol{f}}(\boldsymbol{y})'\left(\boldsymbol{D} + \boldsymbol{D}\boldsymbol{R}_c\boldsymbol{D}'\right)^{-1}\bar{\boldsymbol{f}}(\boldsymbol{y}) = \frac{1}{2}\bar{\boldsymbol{f}}(\boldsymbol{y})'\boldsymbol{Q}_c\bar{\boldsymbol{f}}(\boldsymbol{y}) + \text{ constants} \,.$$

The expression for the posterior is then rewritten as

$$\log P(c|\boldsymbol{y}) \;=\; \log \pi_c + \frac{1}{2}\log\det\boldsymbol{U}_c + \frac{1}{2}\bar{\boldsymbol{f}}(\boldsymbol{y})'\boldsymbol{Q}_c\bar{\boldsymbol{f}}(\boldsymbol{y}) - \frac{1}{2}\boldsymbol{m}_c'\Theta_c\boldsymbol{m}_c + \bar{\boldsymbol{f}}(\boldsymbol{y})'\boldsymbol{U}_c\boldsymbol{m}_c + \text{ constants} \,.$$

REFERENCES

Petersen, K. B. and Pedersen, M. S. (2008), The Matrix Cookbook, *Technical University of Denmark*, 7–15
